# Researches on the Origin and Conditions of Existence of Eliptiform Convulsions, Following Hemorrhage, and on Epilepsy in General

**Published:** 1858-05

**Authors:** A. Kussmaul, A. Tenner


ARTICLE VIII.

TRANSLATED BY THOMAS BEVAN, M. D.

### RESEARCHES ON THE ORIGIN AND CONDITIONS OF EXISTENCE OF EPILEPTIFORM CONVULSIONS, FOLLOWING HEMORRHAGE, AND ON EPILEPSY IN GENERAL.—BY A. KUSSMAUL AND A. TENNER.

The authors of these important researches have taken as a motto the following phrase of Esquirol: "Let us confess frankly that the progress of pathological anatomy, up to the present time, has shed no light upon the immediate seat of epilepsy. It must not, however, discourage us; nature will not always rebel against the efforts of her investigators." * The authors believe to have found the solution of the principal questions relative to epilepsy. I am not entirely of their opinion. I believe, on the one hand, that some of the solutions they publish do not appertain to them, for I had arrived at the same conclusions before; on the other, some of the solutions they propose appear to me erroneous or insufficiently demonstrated. However it may be, they have at least the merit of having approached with profound physiological knowledge (which was indispensable) the study of the phenomena of epilepsy, and of having sought to resolve the numerous questions relating to them, by the aid of rational experiments, and from the comparison of experimental facts with the pathological facts observed in man, I hasten to say, if they have emitted as "new" opinions which I had already proposed, it was evidently because they were unacquainted with my publications in this relation.

Want of space will oblige me to limit myself to the conclusions of Kussmaul and Tenner, adding simply a few remarks.

"1. The convulsions which supervene in cases of hemorrhage in warm blooded animals and in man, are similar to those observed in epilepsy."

This is not a new opinion. In old times, physicians have noted this analogy; and of late years, Dr. C. Bland Radcliffe has in-

* "Avouons franchement que les progres de l'anatomie pathologique n'ont jusqu'ici repandu aucune lumiere sur le siege immediat de l'epilepsie, cependant il ne faut pas se decourager; la nature ne sera pas toujours rebelle aux efforts de ses investigateurs."

sisted on this resemblance, because it was of much importance for his theories of muscular contractions and epilepsy.

"2. The same form of convulsions is manifested when we suddenly prevent the passage of red blood to the encephalon, by ligating the large arterial trunks at the neck."

"3. Epileptiform convulsions likewise occur when the arterial blood takes the character of venous, as after ligation of the trachea."

Marshall Hall showed a long time ago the resemblance between convulsions due to strangulation and those of epilepsy. What remained to be done in this relation was to show why asphyxia, why loss of blood, and why ligation of the carotid and vertebral arteries determined convulsions similar to those of epilepsy.

"4. It is very probable that the production of convulsions in those different cases depends upon the sudden interruption of the nutrition of the brain. This production does not depend on the changes of pressure which occur in the encephalon."

This is one of the fundamental points of the theory of Kussmaul and Tenner. They have arrived by the aid of very interesting experiments to emit an opinion which very much resembled those of several English physicians, and particularly those of Dr. J. R. Reynolds. For myself, I do not deny that a change of nutrition in the brain might engender an agent of excitation which, stimulating this organ, could determine epileptiform convulsions, but I believe it is much more probable that these convulsions are due to excitation caused by principles which are produced in much more than ordinary quantity in the stagnated blood filling the capillaries of the encephalon. The only one of these principles that I know, and perhaps no others exist, is carbonic acid. I have given this view of the subject in my book on epilepsy.*

"5. The epileptiform convulsions due to hemorrhage do not proceed from the medulla spinalis."

---

* Researches on epilepsy, its artificial production in animals, and its etiology, nature, and treatment in man, Boston, 1856–7. These researches were first published in the *Boston Med. and Surg. Journal*, from Nov. 1856 to Oct. 1857. Several of the new theories there detailed had already been emitted in a communication that I made to the Med. Society of the XII. Arrondissement of Paris in Oct. 1856.

This proposition is not correct, which has misled the excellent observers whose ideas I expound; it is true in rabbits, animals upon which they have especially experimented. There is scarcely a trace of convulsive movement, where, after having cut the spinal cord across, we determine a hemorrhage, or when we suppress the circulation in the spinal column without cutting it, by the proceeding that Kussmaul and Tenner indicate; but in other animals, the sheep, according to Marshall Hall, the Guinea pig, birds, and even, but to a less degree, in cats, and also sometimes in dogs. After what I have observed, there are considerations where the spinal cord suddenly ceases to receive blood, especially if it be separated from the cervical region. To render the proposition of the authors we criticise strictly true, we must say the epileptiform convulsions due to hemorrhage do not proceed but in small part from the medulla spinalis.

"6. These convulsions are not due to the brain."

I have demonstrated, in a manner differing from Kussmaul and Tenner, the exactitude of this proposition, having removed the brain from epileptic animals, and the attack continued almost as before.

"7. The central seat of the convulsions is especially to be sought in the parts of the encephalon posterior to the thalami opticorum."

This results also from my own experiments.

"8. Anæmia of the parts of the brain anterior to the crura cerebi in man produces loss of consciousness, insensibility, and paralysis. When it is joined to convulsions, it ought to be attributed to alterations of the excitable parts behind the optic thalami."

I had tried to establish before Kussmaul and Tenner these two fundamental propositions: first, that in epileptic vertigo with or without convulsions the vessels of the cerebral lobes contract and chase the blood from this organ, and in consequence determine in it the condition present in syncope; second, that the cranium does not permit sensible atmospheric pressure to act upon the brain, it suffices that the cerebral lobes receive less blood, for the base of the encephalon to receive more, which for several reasons that I have mentioned in my work on epilepsy, contributes to the production of convulsions.

"9. Anæmia of the spinal cord produces paralysis of the members of the respiratory muscles and those of the neck. If the diminution in the quantity of blood be sudden and considerable, there is sometimes but rarely tremors in the limbs preceding the paralysis. The sphincter of the anus comports itself similar to the sphincter of the eye; at the time of the anæmia of the brain, it contracts convulsively before relaxation."

I repeat, the convulsions are less strong in rabbits than in other animals.

"10. Convulsions in cases of hemorrhage are not due either to a moral cause or to a reflex influence."

I have tried to demonstrate that these convulsions were especially due either to excitation by carbonic acid, aside from the reflex influence of the cephalo-rachidien center, and in this my conclusion differs from that of the authors I criticise.

"11. Convulsions from loss of blood do not occur.

"*a.* In cold-blooded animals (at least in frogs).

"*b.* When the loss of blood is made slowly, so that the forces are gradually exhausted.

"*c.* When the animals are very feeble.

"*d.* When the nutrition of the spinal cord has been impaired.

"*e.* When large portions of the encephalon have been extirpated.

"*f.* In etherized animals.

"Without doubt, also, when the excitable parts of the brain have undergone some pathological modification."

It belongs entirely to Kussmaul and Tenner to have determined the influence of several of these circumstances, but they have not explained how this influence is produced. I will soon show that this explanation is very easily given, by admitting, on the one hand, that it is carbonic acid which excites the convulsions, and on the other, that it is necessary to have a sufficient quantity of the excitant, and a certain degree of excitability, that the convulsions may be produced.

"12. The brain in warm-blooded animals cannot be deprived of blood but for a short time without losing the power of re-establishing its functions under the influence of the nourishing fluid, and apparent death is transformed into veritable dissolution. The encephalon in rabbits has preserved this power during two minutes."

It is very true what Kussmaul and Tenner believe impossible. It is in general for the dark blood, but for the red blood it is otherwise. (See the above proposition in my memoir on the properties of the blood, p. 117 to 126).

"13. It sometimes happens, after ligature of the vessels of the neck, that the muscles of the trunk and members die, and cadaveric rigidity manifests itself before the movements of the left side of the heart cease. The left side of the heart is not always therefore the *primum moriens* of the muscular system."

A long time ago I published facts analogous to these. I showed that cadaveric rigidity occurred in the posterior parts of a living animal, after ligation of the aorta. (*Comptes rendus de l'Academie des Sciences*, 1851, Vol. XXXII., p. 855 et p. 897). I had already seen cadaveric rigidity manifested in the face, neck, and members, while the heart was yet beating. (*Comptes rendus de la Societe de Biologie*, 1re *Serrie*, Vol. II. 1850, p. 154.

"14. The contraction followed by dilatation of the pupils during the agony is not a certain sign that death will inevitably follow, and that the return of life is impossible, as was believed by Bouchut."

"15. For the cure of convulsions occasioned by the absence of blood, there is no means more suitable than the return of the red blood."

"16. The antiphlogistic method (affaiblissante), and especially bleedings, ought to be always rejected in the treatment of epilepsy."

"17. The quantity of blood in the cranial cavity during life can be notably augmented or diminished by the aid of experiments."

"18. The quantity of blood is augmented in the cranial cavity, when the arterial current is allowed to re-establish itself, after having interrupted it by ligating the large arteries of the neck (arterial congestion), when we ligate the veins of the neck (venous congestion), also when we cut the two cords of the great sympathetic nerve at the neck (arterial and venous congestion), lastly, when we tie the trachea during inspiration (venous congestion by asphyxia)."

The preceding propositions are not strictly correct. Certainly, the quantity of blood in the brain may vary, but only within very restricted limits. It is certain also that the quantity of blood can be notably augmented in *one part* of the cranial cavity, but only on the condition of a diminution in another part, which takes place especially when the great sympathetic is cut on both sides at the neck.

"19. Diminution of the quantity of blood in the cranial cavity is produced by hemorrhage and ligation of the arteries at the neck (passive anæmia), by galvanic irritation of the nerves of the vessels of the head (active anæmia)."

The galvanization of the great sympathetic nerve, as Callenfels and Donders have found, and as I have myself often seen, only acts upon certain bloodvessels, and the diminution of the quantity of blood in this case, as in the others (hemorrhage and ligature), is only local, and cannot operate when the cranium is not open, without there being augmentation in other parts, so that the anæmia of one part is accompanied by congestion of another.

"20. There is more blood contained in the cranial cavity after ligature of the arteries than after hemorrhage. The diminution of the quantity of blood always occurs equally in the small arteries, capillaries, and the smallest veins."

The small vessels generally empty themselves more than the medium and large veins and sinuses, which contain more blood as the small vessels are more empty.

"21. It is rarely possible to draw a just conclusion of the quantity of blood that exists in the cranial cavity during life by what is found after death. The last agony is accompanied by numerous circumstances which modify the current of the blood in the cranium, and it is possible, changes in its quantity operate even after death."

Changes in the absolute quantity of blood may certainly occur; the quantity of the cephalo-rachidien fluid may be slightly modified; but atmospheric pressure, being almost without effect on the encephalon, the variations in the quantity of blood in the interior of the cranium cannot be considerable. Displacements may however occur, and one part may empty itself while another becomes exceedingly distended.

"22. The phenomena of the incomplete epileptic access proceeds from alteration of the brain, but those of the complete attack arise from alterations of the entire encephalon. Epileptic convulsions are rationally considered as in their source encephalic, and the spinal cord has probably only the *role* of a conductor who transmits to the muscles the excitations coming from the encephalon.

I have shown that vertigo, loss of consciousness, and insensibility, depend from the cerebral lobes not receiving blood, or not enough of it. As to the spinal cord, I have tried to show that it is not the principal source of the excitations which cause the muscular contractions, but that, at the period of asphyxia of the complete attack, it participates in the production of clonic convulsions; consequently, it is erroneous with Kussmaul and Tenner to attribute to it simply the part of a conductor.

"23. The anatomical alterations that have been described, or those most persistant, cannot be considered as the immediate cause of epileptic attacks, but only as circumstances that predispose to epilepsy."

This is what I have tried to demonstrate by chemical and experimental facts.

"24. Pathological anatomy cannot conduct us to any conclusion with regard to the nature of epilepsy."

"25. The sudden alteration of nutrition which modifies the normal condition of the encephalon, is not manifested until the moment of the attack."

These conclusions also result from my own researches.

"26. The arterial congestion does not appear to be capable of determining anything but the phenomena of paralysis (vertigo and apoplexy)."

This proposition harmonizes with the opinion emitted by myself, that it is especially carbonic acid that causes the convulsions in troubles of the circulation of the encephalon.

"27. Venous congestion of the encephalon, as well as arterial and venous congestion, engenders conditions which before being apoplectic are epileptic, and are characterized by reason of the paralysis of the glottis by a notable abasement of the respiration, and by less violent convulsive phenomena."

I avow I do not understand what Kussmaul and Tenner would say by this. Paralysis of the glottis never arrives as the initial phenomenon, and they do not explain how it can diminish the respiration and convulsions.

"28. It is not the *sphagiasmus* nor the *trachelismus* of Marshall Hall, but the *laryngismus* that must be accused as the source of that epileptic access. All the theories which make the epileptic attack result from a sudden afflux of blood, be it active, passive, or mixed, are false."

The trachelismus can contribute to augment the violence of an attack the same as all the other causes of cerebral congestion. The laryngismus plays a very great part in the production of an attack, but I believe I have demonstrated that it would not be true to admit that the principal part belongs to it. I have shown, besides, that spasm of certain respiratory muscles of the thorax have sometimes a greater part than that of the larynx in the production of epileptiform convulsions.

"29. It is probable that certain forms of epilepsy consist only in convulsions of the muscular walls of the vessels of the brain."

Before Kussmaul and Tenner I had endeavored to demonstrate, basing myself on the same experimental facts that they report, and others also, that certain forms of epilepsy, such as vertigo, etc., depend on contractions of the vessels of the cerebral lobes.

"30. The state that predisposes to the epileptic attack exists sometimes in the entire encephalon, sometimes in one part only, which produces in the rest an impaired condition that is the foundation of the epileptic attack."

I have only to say to this proposition, that I have tried to establish that the particular state that constitutes the predisposition to the epileptic attack does not exist in the cerebral lobes, but in the parts which constitute the isthmus of the encephalon, adding perhaps a portion of the cervical cord.

"31. The medulla oblongata appears, inasmuch as it is the origin of the nerves of the constrictors of the glottis and the vaso-motory nerves, to be most ordinarily the point of origin of eclampsic and epileptic attacks."

I had already emitted this opinion, but in adding that the protuberance, and perhaps a part of the spinal cord, ought also to be regarded as points of origin of the convulsive attacks, not only because, according to my researches, they gave birth to the vaso-motory nerves, but for other reasons that may be found in my book on epilepsy.

E. BROWN-SEQUARD,

*Journal de la Physiologie de l'Homme et des Animaux*

